# Human intrahepatic regulatory T cells are functional, require IL‐2 from effector cells for survival, and are susceptible to Fas ligand‐mediated apoptosis

**DOI:** 10.1002/hep.28517

**Published:** 2016-04-15

**Authors:** Yung‐Yi Chen, Hannah C. Jeffery, Stuart Hunter, Ricky Bhogal, Jane Birtwistle, Manjit Kaur Braitch, Sheree Roberts, Mikaela Ming, Jack Hannah, Clare Thomas, Gupse Adali, Stefan G. Hübscher, Wing‐Kin Syn, Simon Afford, Patricia F. Lalor, David H. Adams, Ye H. Oo

**Affiliations:** ^1^ Centre for Liver Research and NIHR Birmingham Liver Biomedical Research UnitUniversity of BirminghamBirminghamUnited Kingdom; ^2^ Institute of Immunology and ImmunotherapyUniversity of Birmingham, BirminghamUnited Kingdom; ^3^ Clinical Immunology DepartmentUniversity Hospital Birmingham NHS Foundation TrustBirminghamUnited Kingdom; ^4^ Department of Cellular PathologyQueen Elizabeth Hospital BirminghamUnited Kingdom; ^5^ The Institute of HepatologyLondonUnited Kingdom; ^6^ Division of Gastroenterology and HepatologyThe Medical University of South CarolinaCharlestonSouth CarolinaUSA

## Abstract

Regulatory T cells (T_reg_) suppress T effector cell proliferation and maintain immune homeostasis. Autoimmune liver diseases persist despite high frequencies of T_reg_ in the liver, suggesting that the local hepatic microenvironment might affect T_reg_ stability, survival, and function. We hypothesized that interactions between T_reg_ and endothelial cells during recruitment and then with epithelial cells within the liver affect T_reg_ stability, survival, and function. To model this, we explored the function of T_reg_ after migration through human hepatic sinusoidal‐endothelium (postendothelial migrated T_reg_ [PEM T_reg_]) and the effect of subsequent interactions with cholangiocytes and local proinflammatory cytokines on survival and stability of T_reg_. Our findings suggest that the intrahepatic microenvironment is highly enriched with proinflammatory cytokines but deficient in the T_reg_ survival cytokine interleukin (IL)‐2. Migration through endothelium into a model mimicking the inflamed liver microenvironment did not affect T_reg_ stability; however, functional capacity was reduced. Furthermore, the addition of exogenous IL‐2 enhanced PEM T_reg_ phosphorylated STAT5 signaling compared with PEMCD8. CD4 and CD8 T cells are the main source of IL‐2 in the inflamed liver. Liver‐infiltrating T_reg_ reside close to bile ducts and coculture with cholangiocytes or their supernatants induced preferential apoptosis of T_reg_ compared with CD8 effector cells. T_reg_ from diseased livers expressed high levels of CD95, and their apoptosis was inhibited by IL‐2 or blockade of CD95. *Conclusion*: Recruitment through endothelium does not impair T_reg_ stability, but a proinflammatory microenvironment deficient in IL‐2 leads to impaired function and increased susceptibility of T_reg_ to epithelial cell‐induced Fas‐mediated apoptosis. These results provide a mechanism to explain T_reg_ dysfunction in inflamed tissues and suggest that IL‐2 supplementation, particularly if used in conjunction with T_reg_ therapy, could restore immune homeostasis in inflammatory and autoimmune liver disease. (Hepatology 2016;64:138–150)

**Abbreviations**
ANOVA: analysis of variance
BEC: biliary epithelial cell
ELISA: enzyme‐linked immunosorbent assay
FASL: Fas ligand
IL: interleukin
HSEC: hepatic sinusoidal endothelial cell
IFN‐γ: interferon‐γ
LIT_reg_: liver‐infiltrating regulatory T cell
PEM T_reg_: postendothelial migrated T_reg_
SEM: standard error of the mean
Th 1: T helper 1
Th 17: T helper 17
TNF‐α: tumor necrosis factor α
T_reg_: regulatory T cell

CD4^+^CD25^+^ CD127^low^ FOXP3^+^ regulatory T cells (T_reg_) maintain peripheral self‐tolerance by suppressing T effector cell proliferation and function.^1^, ^2^ T_reg_ depletion or dysfunction leads to autoimmunity in mice and IPEX syndrome in humans. T_reg_ dysfunction or an imbalance between T_reg_ and effector cells in tissue will determine the outcome of inflammation.^3^, ^4^ Intrahepatic T_reg_ increase in parallel with effector T cells during chronic hepatitis^5^ demonstrates that the presence of T_reg_ in the liver does not prevent ongoing hepatic inflammation. Whether this is due to dysfunctional T_reg_ or overwhelming effector responses is not known.

Understanding the fate of T_reg_ in the inflamed liver is crucial, because T_reg_ recruited to the liver from blood via hepatic sinusoids^6^ enter the hepatic microenvironment enriched with proinflammatory cytokines. The process of recruitment and/or exposure to this inflammatory microenvironment could affect T_reg_ stability, survival, and function. Recruitment through endothelium can affect the differentiation and activation of various leukocyte subsets, and T_reg_ can switch toward a T helper 1 (Th 1) or T helper 17 (Th 17) lineage in an inflammatory environment.^7^, ^8^ Furthermore, intrahepatic lymphocytes are removed by activation‐induced cell death triggered by receptors such as CD95 (APO‐1/Fas), leading to resolution of inflammation. Thus, differential susceptibility to apoptosis could affect the balance of T_reg_ and T effector cells at inflammatory sites.^9^, ^10^ T_reg_ are highly sensitive to CD95‐mediated apoptosis,^11^ and interleukin (IL)‐2‐dependent phosphorylation of STAT5 is crucial for their proliferation, differentiation, and survival.^12^ We reported previously that many intrahepatic T_reg_ lack evidence of STAT5 phosphorylation, suggesting that the inflammatory liver microenvironment is hostile to T_reg_ survival and function.^13^

In the present study, we report that recruitment through endothelium into a model of the inflamed liver microenvironment reduces the suppressive capacity of T_reg_ and a lack of local IL‐2 enhances their susceptibility to Fas‐mediated apoptosis induced by epithelial cells. These findings support the therapeutic potential of IL‐2 therapy to restore local T_reg_ function in inflammatory liver diseases.

## Materials and Methods

### TISSUES AND BLOOD

Venous blood was obtained from healthy volunteers or patients with hemochromatosis who were admitted to the Liver Unit at the Queen Elizabeth Hospital, Birmingham, UK^5^ (Local Research Ethics Committee reference no. 04/Q2708/41) and liver tissues from patients undergoing liver transplantation for inflammatory liver diseases, including primary biliary cirrhosis, primary sclerosing cholangitis, alcoholic liver disease, and autoimmune hepatitis. Normal liver was obtained from donor liver tissue surplus to clinical requirements (Local Research Ethics Committee reference no. 06/Q2708/11, 98/CA5192).

### ISOLATION OF LIVER INFILTRATING LYMPHOCYTES, BILIARY EPITHELIAL CELLS, AND PRIMARY HUMAN HEPATIC SINUSOIDAL ENDOTHELIAL CELLS

Liver‐infiltrating lymphocytes,^5^ biliary epithelial cells (BECs),^14^ and hepatic sinusoidal endothelial cells (HSECs)^15^ were prepared and isolated from fresh liver tissue as described previously.^13^

### POSTENDOTHELIAL TRANSMIGRATED T_reg_ AND CD8^+^ T CELL FUNCTIONAL ASSAYS

HSECs were plated and grown on six‐well collagen I precoated Transwell inserts (3 μm; Greiner Bio‐One) until confluent. Cells were then stimulated with 10 ng/mL interferon‐γ (IFN‐γ) and 10 ng/mL tumor necrosis factor α (TNF‐α) (both Peprotech) for 24 hours to mimic the inflamed environment. After washing, the lower chamber was filled with either Roswell Park Memorial Institute 1640 (RPMI‐1640) medium (Gibco) supplemented with 0.05% bovine serum albumin or inflamed liver conditioned supernatant prepared by incubating 1 g of tissue in 4 mL serum‐free RPMI 1640 for 24 hours. The supernatant was then centrifuged and filtered (0.22 μm‐pore). Freshly isolated T_reg_ or CD8^+^ T cells were then added to the upper chamber of the Transwell insert in 0.05% bovine serum albumin/RPMI‐1640 and allowed to migrate across the endothelial cell monolayer into the lower chamber for 24 hours. The postendothelial transmigrated T_reg_ (PEM T_reg_) or postendothelial transmigrated CD8^+^ T (PEM CD8) cells were collected for experiments (T_reg_ suppression assay, biliary coculture assay, phosphorylated STAT5 assay, and T_reg_ plasticity assay).

### PEM T_reg_ AND CD8 APOPTOSIS ASSAY

BECs were seeded into 24‐well collagen I precoated plates, grown until confluent, and stimulated with 10 ng/mL IFN‐γ and 10 ng/mL TNF‐α (Peprotech) for 24 hours. PEM T_reg_ or PEM CD8 cells were cocultured with these BECs or with BEC supernatant for 24 hours, and apoptosis was analyzed by way of flow cytometric staining using Annexin and 7AAD (BD Pharmingen).

### STATISTICAL ANALYSIS

Differences between treatments were evaluated by way of Student *t* test or one‐way analysis of variance (ANOVA), followed by Bonferroni multiple comparison test using GraphPad Prism version 6.0 (GraphPad Software). *P* < 0.05 was considered statistically significant. Data are presented as the mean ± standard error of the mean (SEM).

See the Supporting Information for details regarding immunohistochemistry, confocal microscopy, enzyme‐linked immunosorbent assay (ELISA), flow cytometry, isolation of peripheral blood T_reg_ and CD8 and assays of T_reg_ function, plasticity, and response to IL‐2.

## Results

### FRESHLY ISOLATED HUMAN LIVER INFILTRATING T_reg_ ARE ACTIVATED, NONEXHAUSTED, MEMORY CELLS

**Figure 1:**
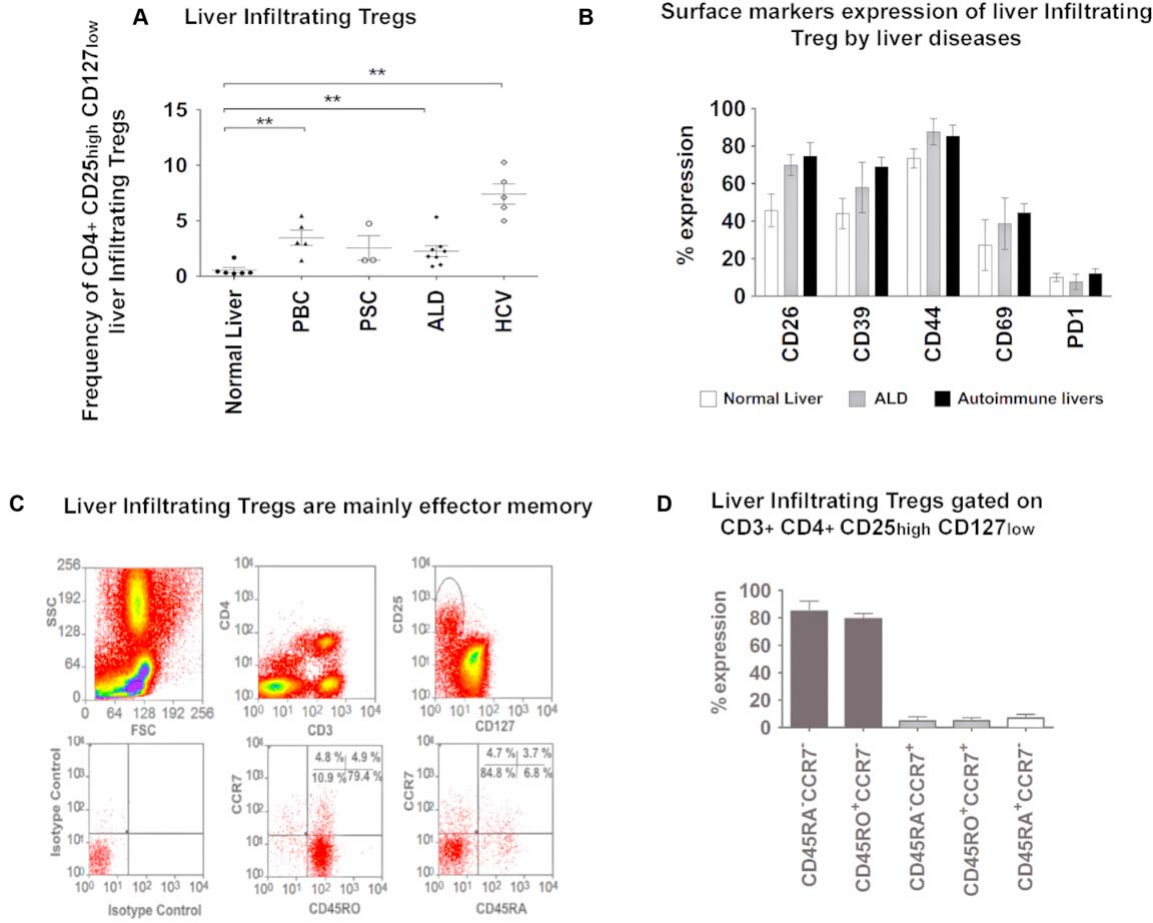
Frequency and phenotype of intrahepatic T_reg_ in inflamed human livers. Freshly isolated LIT_reg_ from human explanted livers were phenotyped by flow cytometry. LIT_reg_ were gated as CD4^+^CD25^high^CD127^low^. (A) Frequency of LIT_reg_ in normal livers and different diseased livers. (B) Surface marker expressions of LIT_reg_ from normal livers, alcoholic liver disease livers, and autoimmune disease livers (see Supporting Fig. S1C for surface marker expressions of LICD8 cells from different diseased livers). LIT_reg_ (defined as CD3^+^CD4^+^CD127^low^CD25^high^) were screened for expression of surface markers, CD26, CD39, CD44, CD69, and PD1. Data are presented as the mean ± SEM (n = 6; one‐way ANOVA followed by Bonferroni multiple comparison test). **P < 0.05. (C, D) Maturation status of LIT_reg_. Frequencies of CD45RA^−^ memory (CD45RA^−^CCR7^−^), CD45RO^+^ memory (CD45RO^+^CCR7^−^), central memory (CD45RA^−^CCR7^+^), and naïve (CD45RA^+^ CCR7^+^) LIT_reg_ populations were determined by way of flow cytometry. Representative dot plots (C) and summary data (D) of LIT_reg_ maturation status are shown. Data are presented as the mean ± SEM (n = 6).

We compared the frequency and surface phenotype of liver‐infiltrating CD4^+^CD25^high^CD127^low^ T_reg_ (LIT_reg_) in different inflammatory liver diseases with normal liver tissue (Fig. 1A,B) and with liver‐infiltrating CD8 cells (Supporting Fig. S1C). T_reg_ isolated from normal and diseased livers differed in their expression of T_reg_‐associated functional surface receptors CD26, CD39, and CD69 (Fig. 1B). Very low expression of PD1 was observed on LIT_reg_ in both normal and diseased patients (9±5% versus 9.5±2%) (Fig. 1B) but high levels of CD44 were observed on LIT_reg_ (79±6% versus 87±5%) and liver‐infiltrating CD8 cells (75±9% versus 90±4%) (Supporting Fig. S1C). LIT_reg_ exhibited a memory phenotype: CD45RO^high^CD45RA^low^ and CCR7^low^ (85±7%) and inflamed liver tissue contained small populations of central memory CD45RA^neg^CCR7^pos^ (4.7±3%) and tissue resident memory CD45RA^pos^CCR7^neg^ (7±3%) LIT_reg_ (Fig. 1C,D).

### PROINFLAMMATORY CYTOKINES ARE ELEVATED IN THE INFLAMED LIVER ENVIRONMENT, BUT THIS DOES NOT ALTER INTRAHEPATIC T_reg_ STABILITY

**Figure 2:**
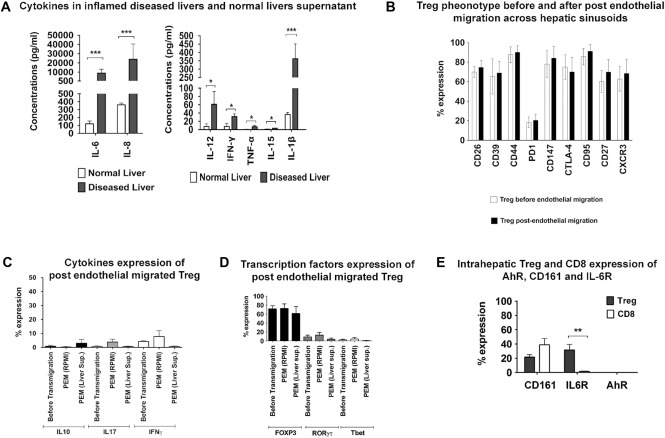
Intrahepatic microenvironment is enriched with proinflammatory cytokines and PEM T_reg_ are not plastic to other T cell lineages. (A) Cytokine profiles of supernatants collected from normal and diseased livers. Tissue from normal or diseased liver was cultured for 24 hours in RPMI‐1640 medium at 1 g/mL, and the concentrations of cytokines IL‐6, IL‐8, IL‐12, IL‐15, IFN‐γ, TNF‐α, and IL‐1β were measured using a Bio‐Plex Pro bead‐based multiplex kit. Data are presented as the mean ± SEM (n = 6; one‐way ANOVA with Bonferroni multiple comparison test). *P < 0.05. (B‐D) T_reg_ phenotype before and after migration across stimulated hepatic sinusoidal endothelium. T_reg_ freshly isolated from peripheral blood were transmigrated through monolayers of IFN‐γ‐ and TNF‐α‐stimulated hepatic sinusoidal endothelial cells into either RPMI‐1640 medium (control) or liver supernatant and phenotyped by way of flow cytometry. Expression of markers including CD26, CD39, CD44, PD1, CD147, CTLA‐4, CD95, CD27, and CXCR3 before and after transendothelial migration into RPMI‐1640 medium are shown (B). Data are presented as the mean ± SEM (n = 6; one‐way ANOVA followed by Bonferroni multiple comparison test). *P < 0.05. Expressions of cytokines including IL‐10, IL‐17, and IFN‐γ at 24 hours (C) and transcription factors FOXP3, ROR, and Tbet at 3 days (D) were measured before and after migration into RPMI‐1640 medium or liver supernatant (see Supporting Fig. S2 for expression of transcription factors up to 3 days of culture in inflamed liver supernatants). Data are presented as the mean ± SEM (n = 4; one‐way ANOVA). *P ≤ 0.05. (E) Expression of CD161, IL‐6 receptor, and intracellular AhR by LIT_reg_ directly ex vivo was shown. Data are presented as the mean ± SEM (n = 6; one‐way ANOVA followed by Bonferroni multiple comparison test). *P < 0.05; **P < 0.01; ***P < 0.005

We explored the intrahepatic microenvironment by measuring proinflammatory cytokines in supernatants from inflamed human liver tissue. Diseased liver supernatants contained higher concentrations of IL‐6 (8960 ± 4257 pg/mL), IL‐8 (24,033 ± 16,589 pg/mL), IL‐12 (61 ± 30 pg/mL), IFN‐γ (32 ± 7 pg/mL), and IL‐1β (363 ± 88 pg/mL) compared with normal liver supernatants (Fig. 2A).

We then examined the stability of intrahepatic T_reg_ in the hepatic microenvironment by modeling recruitment and intrahepatic conditions in vitro. Blood T_reg_ that had transmigrated across TNF‐α and IFN‐γ‐stimulated HSECs had a phenotype similar to the LIT_reg_ phenotype, allowing us to use them to model the inflamed liver in vitro (Fig. 2B). PEM T_reg_ were migrated across stimulated HSECs into either control media or supernatants from inflamed liver tissue cultures (Supporting Fig. S5) and secretion of IL‐10, IL‐17, and IFN‐γ by T_reg_ measured 24 hours postmigration (Fig. 2C) and expression of FOXP3, ROR, and Tbet analyzed up to 3 days (Fig. 2D, Supporting Fig. S2). A subset of LIT_reg_ expressed CD161 (20%) or IL‐6 receptor (27%). We did not detect AhR expression (Fig. 2E).

### PEM T_reg_ REMAIN FUNCTIONALLY SUPPRESSIVE

**Figure 3:**
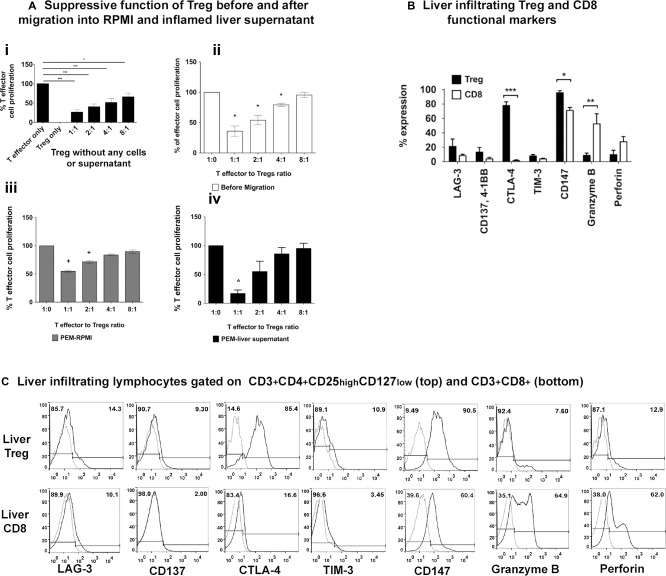
Intrahepatic T_reg_ remain functional and express T_reg_ functional markers. (A) T_reg_ representative of intrahepatic T_reg_ phenotype were generated by transmigration of freshly isolated blood T_reg_ across TNF‐α‐ and IFN‐γ‐stimulated human sinusoidal endothelium into RPMI‐1640 control medium or inflamed liver supernatant for 24 hours. The suppressive function of T_reg_ was analyzed by testing their ability to suppress the proliferation of CellTrace Violet prestained CD4 T effector cells using Miltenyi T_reg_ suppression inspector. T_reg_ populations compared included: (i) T_reg_ directly isolated, without contact with any cells or supernatant; (ii) T_reg_ in contact with endothelium but not transmigrated; (iii) PEM T_reg_ migrated into control RPMI media; and (iv) PEM T_reg_ migrated into liver supernatant. (B) Comparison of the expressions of T_reg_ functional markers by freshly isolated human LIT_reg_ and CD8 T cells. Data are presented as the mean ± SEM (n = 5; one‐way ANOVA followed by Bonferroni multiple comparison test). *P < 0.05; **P < 0.01; ***P < 0.005. (C) Representative flow cytometry overlays for each functional marker on LIT_reg_ (CD3^+^CD4^+^CD25^high^CD127^low^) (top panel) and LICD8 (CD3^+^CD8^+^) (bottom panel).

After migration across endothelium into inflamed tissue supernatants, T_reg_ maintained their ability to suppress the proliferation of T effector cells (Fig. 3A, Supporting Fig. S5A). Freshly isolated T_reg_ were functionally suppressive (T_reg_/T effector cell ratio = 1:8) before any cell contact (Fig. 3Ai) and after contact with endothelium (T_reg_/T effector cell ratio = 1:4) but before transmigration (Fig. 3Aii). After transmigration across TNF‐α and IFN‐γ‐stimulated HSECs into control media (Fig. 3Aiii), T_reg_ had a reduced suppressive capacity at a 1:2 ratio. This was reduced further after migration into inflamed liver supernatant with suppression only seen at a 1:1 cell ratio (Fig. 3Aiv).

LIT_reg_ expressed cell surface receptors associated with suppressive function (Fig. 3B,C) including CTLA‐4 and CD147,^16^ whereas factors associated with cytolytic activity, granzyme‐B, and perforin were expressed at higher levels on LICD8 T cells compared with LIT_reg_ (53% versus 8% and 27% versus 8%). There were no significant differences in the expression of LAG‐3 (21% versus 8%), CD137 (13% versus 4%), and TIM‐3 (8% versus 4%) between LIT_reg_ and LICD8 (Fig. 3B,C).

### LIVER RESIDENT T_reg_ AND CD8 RESIDE AROUND Fas LIGAND (FASL)‐POSITIVE BILE DUCTS

**Figure 4:**
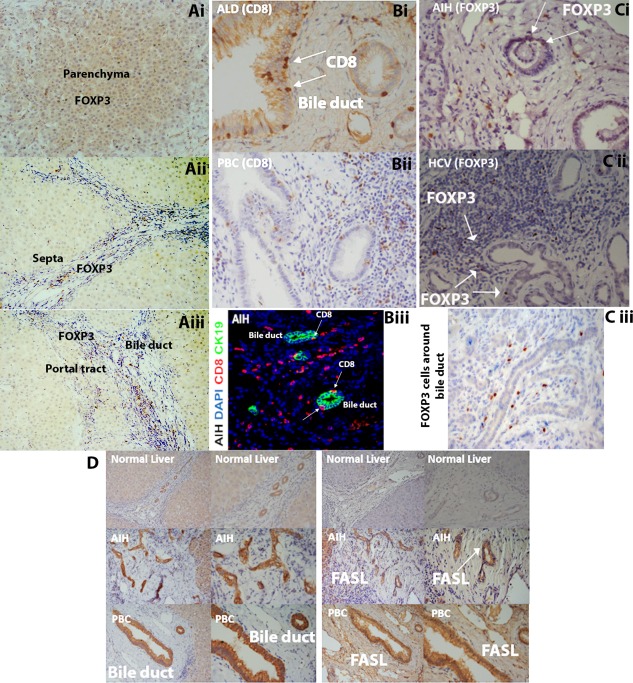
Human diseased liver bile ducts expressing FASL and LIT_reg_ are present across liver lobules, with some residing in the peribiliary region. Immunohistochemistry (A‐D) and confocal fluorescence staining (Biii) of paraffin‐embedded human liver sections for FOXP3, CD8, CK19 (biliary epithelial cell marker), and FASL are shown. (A) intrahepatic FOXP3^+^ T_reg_ are present across the parenchyma (i), septa (ii), and portal tract (iii). (B) LICD8^+^ T cells reside close to bile ducts in the portal tracts in diseased human liver sections (Bi) alcoholic liver disease (ALD), (Bii) primary biliary cirrhosis (PBC), (Biii) Localization of CD8 cells on bile ducts in AIH liver tissue (red = CD8 [phycoerythrin]; green = bile ducts/CK19 [fluorescein isothiocyanate]; blue = nuclear stain [4′,6‐diamidino‐2‐phenylindole]). (C) Localization of FOXP3^+^ cells around the bile duct in diseased livers (Ci) autoimmune hepatitis (AIH), (Cii) hepatitis C virus (HCV), (Ciii) non A non B seronegative hepatitis. (D) Expression of FASL (right panel) by human CK19‐expressing bile ducts (left panel) in normal and diseased livers.

FOXP3^+^ LIT_reg_ were detected throughout the hepatic parenchyma, fibrous septa, and sinusoids as well as close to bile ducts in portal tracts (Fig. 4A‐C) in inflamed liver tissue. CD95 ligand (FASL/CD178) was detected on CK19^+^ bile ducts in all inflammatory liver disease but not on normal liver tissue (Fig. 4D),^14^ with no difference in expression between different liver diseases.

### CD95 EXPRESSION IS INCREASED ON LIVER INFILTRATING T_reg_ AND MEDIATES CELL APOPTOSIS IN RESPONSE TO BEC FASL

**Figure 5:**
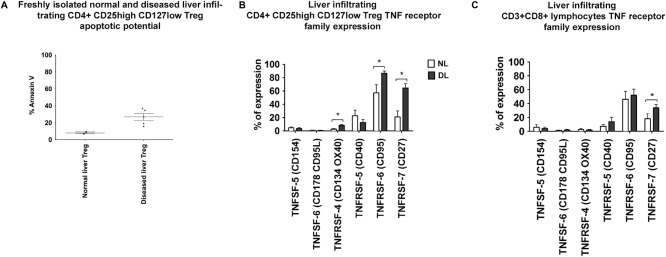
Apoptotic potential and expression of the TNF receptor CD95 is increased on LIT_reg_ in diseased livers. (A) The apoptotic potential of LIT_reg_ in both normal and diseased livers was assessed by way of flow cytometry with Annexin 5 staining directly ex vivo. (B, C) Expression of TNF superfamily (TNFSF) and TNF receptor superfamily (TNFRSF) markers by LIT_reg_ (CD3^+^CD4^+^CD127^low^CD25^high^) (B) and liver‐infiltrating CD8 (CD3^+^CD8^+^) (C) in normal livers (white bar = normal liver) and diseased livers (black bar = diseased liver). Data are presented as the mean ± SEM (n = 6; one‐way ANOVA followed by Bonferroni multiple comparison test). *P < 0.05.

**Figure 6:**
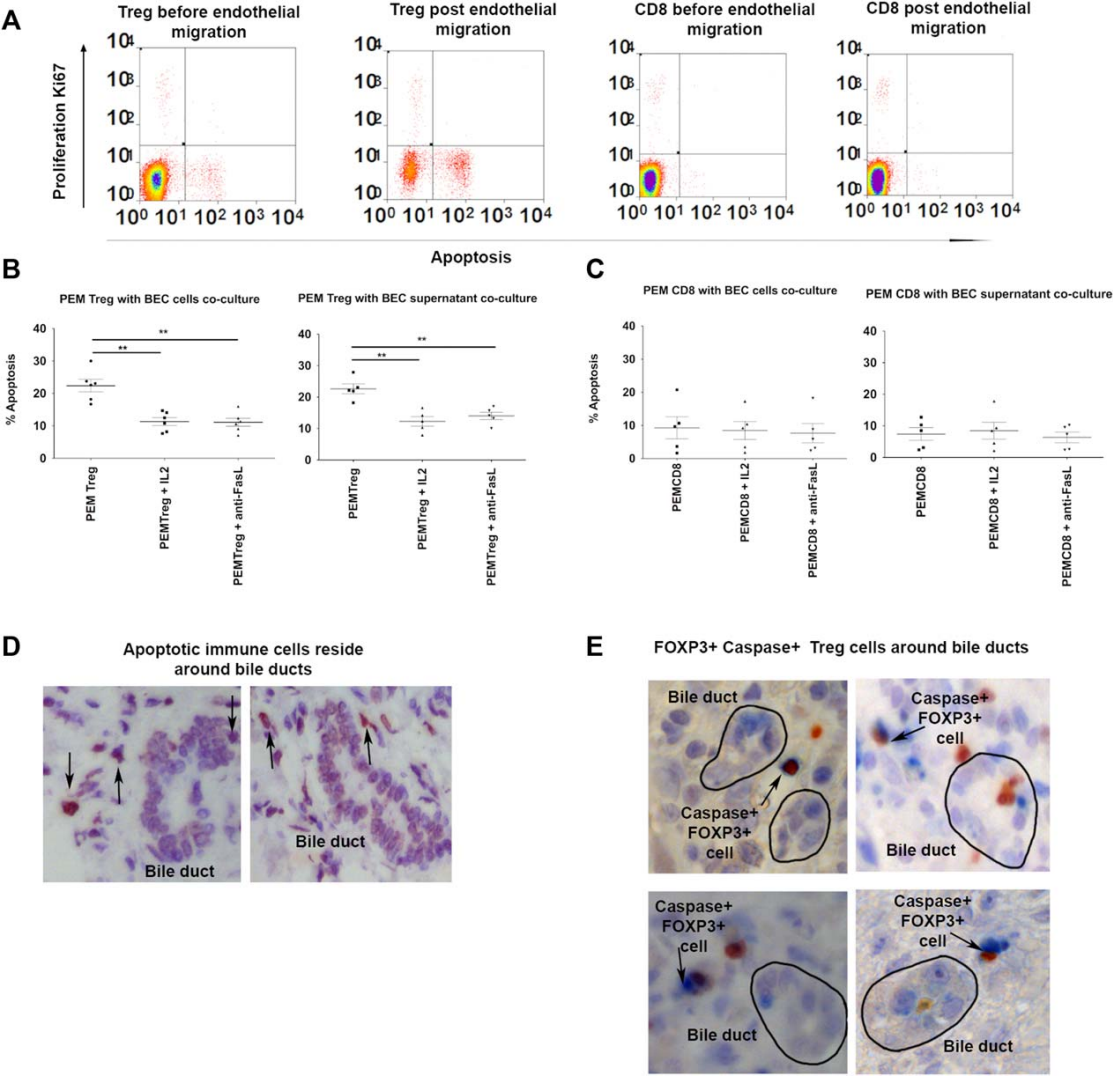
Apoptosis of PEM T_reg_ but not CD8 is FASL dependent and can be rescued by blocking FASL or by supplementing with IL‐2. T_reg_ and CD8+ T cells were transmigrated across TNF‐α‐ and IFN‐γ‐stimulated human sinusoidal endothelium to obtain postendothelial migrated cells, which were then cocultured with BECs or BEC supernatant in the presence or absence of recombinant IL‐2 or anti‐FASL antibody. Apoptosis was analyzed using flow cytometry for Annexin V. (A) Representative dot plots of apoptosis for T_reg_ and CD8 before and after endothelial migration. (B, C) Apoptosis of PEM T_reg_ (B) and PEM CD8 cells (C) with either BECs or BEC supernatant in the presence or absence of IL‐2 or anti‐FASL (one‐way ANOVA followed by Bonferroni multiple comparison tests). **P ≤ 0.05. (D) Terminal deoxynucleotidyl transferase‐mediated deoxyuridine triphosphate nick‐end labeling revealed that apoptotic immune cells were present around bile ducts. (E) Dual staining of FOXP3 (brown) and caspase‐3 (blue) in different diseased livers.

T_reg_ from diseased livers were more susceptible to apoptosis (Fig. 5A). LIT_reg_ expressed CD154, OX40, CD40, CD95, and CD27 and high levels of CD95 in all diseased livers (Fig. 5B). Liver‐infiltrating CD8 cells were expressed at lower levels (Fig. 5C) Freshly isolated T_reg_ and CD8 cells were transmigrated across TNF‐α and IFN‐γ‐stimulated hepatic sinusoidal endothelium into either control media, stimulated BEC supernatant, or contact with BECs (Fig. 6, Supporting Fig. S5B). T_reg_ underwent apoptosis in this model, whereas very few CD8 T cells did. T_reg_ apoptosis in response to contact with BECs or exposure to BEC supernatant was prevented by blocking CD95 ligand or by the addition of IL‐2 (Fig. 6). Apoptotic immune cells were present around the bile duct, and we observed FOXP3 T_reg_ and caspase‐3 dual‐positive apoptotic cells in peribiliary regions in tissue slides (Fig. 6C).

We detected high expression of CD27 on LIT_reg_ and CD70 the ligand for CD27 on liver‐infiltrating dendritic cells. However, recombinant CD70 had no effect on T_reg_ or CD8 T cell proliferation (Supporting Fig. S4).

### EFFECTOR T CELLS ARE THE MAIN SOURCE OF INTRAHEPATIC IL‐2 AND T_reg_ RESPOND BY PHOSPHORYLATION OF STAT5

**Figure 7:**
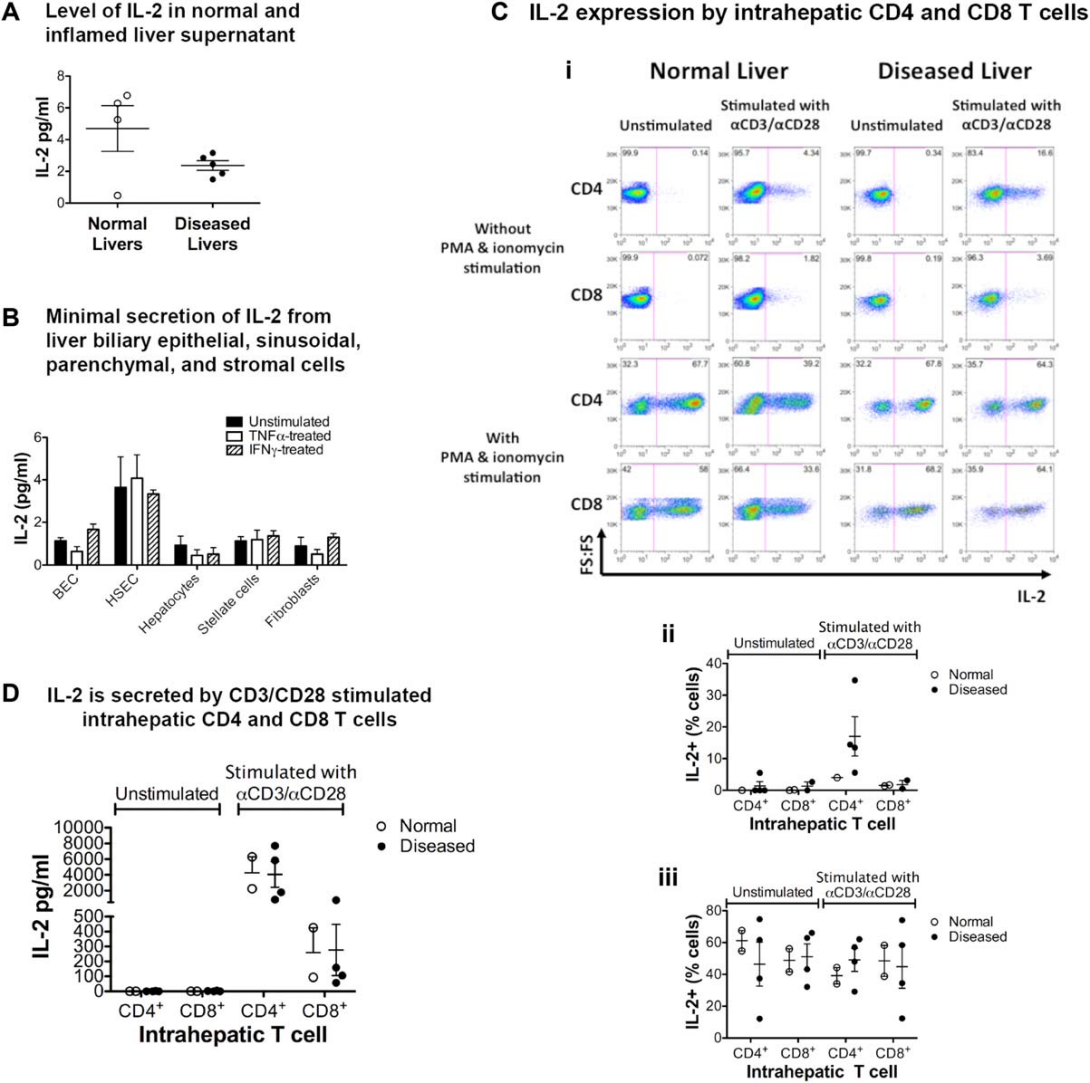
IL‐2 is deficient in the liver microenvironment, and activated T lymphocytes are the source of IL‐2. (A) IL‐2 concentrations in normal and inflamed liver tissue supernatants are shown. Chronic diseased and normal liver tissues were diced and cultured for 24 hours in RPMI‐1640 medium at 1 g/mL, and the concentration of IL‐2 was measured by way of ELISA. (B) Minimal IL‐2 synthesis by liver‐resident cells under normal and inflamed conditions. Cultures of primary BECs, HSECs, hepatocytes, stellate cells, and fibroblasts from human livers were treated with the inflammatory cytokines TNF‐α (10 ng/mL) and IFN‐γ (100 ng/mL) or left untreated, and the concentration of IL‐2 in 24‐hour supernatants was determined by way of Luminex. Data are presented as the mean ± SEM (n = 3). (C, D) Intrahepatic CD4^+^ and CD8^+^ T cells express and secrete IL‐2 upon T cell receptor activation. CD4^+^ and CD8^+^ T cells were isolated from normal and diseased human liver tissues by way of cell sorting and were cultured for 12 hours at 100 cells/mL with or without anti‐CD3/anti‐CD28 stimulation 4 cells/bead. (C) IL‐2 expression was examined by way of flow cytometry. (Ci) Representative flow cytometry dot plots for IL‐2 production by stimulated (CD3/CD28 beads) or unstimulated CD4 and CD8 T cells in the presence or absence of additional PMA and ionomycin stimulation. (Cii, Ciii) Summary frequencies for IL‐2 expression without (Cii) and with (Ciii) additional PMA and ionomycin stimulation. (D) IL‐2 secretion was examined by way of ELISA. PMA, phorbol 12‐myristate 13‐acetate.

IL‐2 was nearly undetectable in supernatants prepared from normal and chronic liver disease tissues (Fig. 7A). Secretion of IL‐2 by human hepatocytes, HSECs, stromal cells, and biliary epithelial cells was minimal (Fig. 7B). IL‐2 expression by liver‐infiltrating activated CD4/CD8 cells was observed by way of flow cytometry (Fig. 7C) and secreted IL‐2 was detected in the supernatant of liver‐infiltrating CD4 and CD8 lymphocytes after stimulation with anti‐CD3/CD28 beads, suggesting that effector lymphocytes are the main source of IL‐2 in inflamed livers (Fig. 7D).

**Figure 8:**
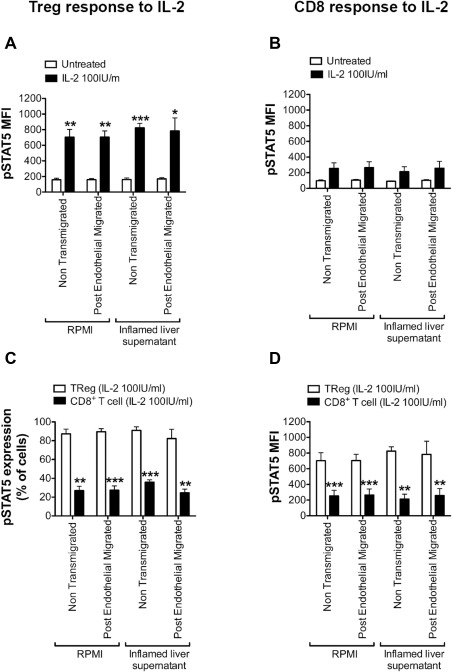
STAT5 phosphorylation is functionally intact in response to supplemented IL‐2 in postendothelial migrated T_reg_ in the liver microenvironment. STAT5 phosphorylation in pre‐endothelial migrated and PEM T_reg_ and CD8 cells in response to IL‐2 stimulation is shown. Peripheral blood mononuclear cells were transmigrated overnight across 24‐hour TNF‐α‐ and IFN‐γ‐stimulated human sinusoidal endothelium into RPMI‐1640 medium or inflamed liver supernatant. Postendothelial migrated cells and non‐transmigrated cells were collected into separate fractions and were stimulated for 10 minutes with 0 or 100 IU/mL IL‐2. (A, B) Phosphorylated STAT5 (pY694) expression by (A) CD4^+^CD25^+^CD127^−^ T_reg_ and (B) CD8 cells in the fractions was examined by way of flow cytometry in the absence of IL‐2 stimulation (untreated) and after 10 minutes of treatment with 100 IU/mL IL‐2. (C) Comparison of the percentages of T_reg_ and CD8 cells expressing phosphorylated STAT5 after treatment with IL‐2. (D) Comparison of the median fluorescence intensity (MFI) of STAT5 phosphorylation in T_reg_ and CD8 cells after treatment with IL‐2. Data are presented as the mean ± SEM (n = 4; paired *t* test). *P < 0.05, **P < 0.01, ***P < 0.0001. Statistical tests compare cells from the same fraction (either untreated and IL‐2 treated T_reg_ or CD8 cells (A and B) or IL‐2 treated T_reg_ and CD8 cells (C and D)).

A high level of STAT5 phosphorylation was induced in 80%‐90% of T_reg_, both non‐transmigrated and PEM T_reg_ (Fig. 8, Supporting Fig. S4), and was not affected by transmigration into the inflamed liver microenvironment (Fig. 8). Significantly fewer CD8 cells (27%‐36%) responded compared with T_reg_ (Supporting Fig. S4), and the level of phosphorylated STAT5 generated was also significantly greater in T_reg_ than in CD8 T cells (Fig. 8). Thus, IL‐2‐CD25 signaling and STAT5 phosphorylation remained intact in T_reg_ after transmigration into the inflamed hepatic microenvironment.

## Discussion

Functionally active T_reg_ are required to suppress effector cells and maintain immune homeostasis,^13^ and the balance of T_reg_ and T effector cells will contribute to the outcome of liver inflammation.^17^ Therapeutic modulation of tissue resident T_reg_ has potential for treating autoimmune liver diseases.^17^, ^18^ T_reg_ enter the liver from blood via hepatic sinusoids^5^ before being localized within the liver in response to signals from the hepatic stromal compartment, hepatocytes, and cholangiocytes.^19^ Once T_reg_ have migrated across the hepatic sinusoid, they are exposed to cytokines including IL‐1β, IL‐6, IL‐8, IL‐12, and IFN‐γ in the inflamed hepatic microenvironment (Fig. 2). This proinflammatory milieu has the potential to influence the stability and function of intrahepatic T_reg_.^20^ We observed that PEM T_reg_ that had migrated through activated endothelium into liver tissue supernatants showed no changes in FOXP3, RORc, or Tbet expression at 3 days, suggesting that T_reg_ differentiation is not affected by the liver milieu. In particular, the lack of ligand‐activated transcription factor aryl hydrocarbon receptor and low levels of CD161, which are associated with Th 17 polarity, suggest few cells differentiate into Th 17 cells within the inflamed environment.^21^, ^22^, ^23^

The frequency of LIT_reg_ is increased across all liver diseases,^24^ and these cells are not exhausted, with very low expression of PD‐1 and high levels of CD69 and maintained functional properties^25^, ^26^ (Fig. 3Aiv). LIT_reg_ exposed to a liver microenvironment maintain high levels of CD39, an ectonucleotidase used by T_reg_ to generate immunosuppressive adenosine from extracellular nucleotides.^27^ Low expression of CD39 has been reported in autoimmune liver disease, and our data suggest that the hepatic microenvironment cannot explain this finding.^28^ Similarly, LIT_reg_ expressed high levels of CD44, which has been associated with FOXP3 expression and suppressive function.^29^ After transendothelial migration into inflamed liver supernatant, T_reg_ maintained IL‐10 secretion and CD147 expression, a marker of activated and suppressive T_reg_.^16^ In addition, we also observed an enrichment of CTLA‐4 expression in LIT_reg_. CTLA‐4 interacts with and removes CD80/CD86 from dendritic cells by way of transendocytosis, resulting in impaired costimulation via CD28 and is thus critical for T_reg_ function.^30^ Overall, LIT_reg_ are equipped with key surface markers and intracellular cytokines required to execute their suppressive capacity.^31^, ^32^ These findings are encouraging for proposed studies of adoptive T_reg_ therapy in autoimmune and inflammatory liver diseases because they suggest that therapeutic cells will remain functional and will not differentiate into effector cells in an inflammatory environment.^17^

We detected LIT_reg_ and LICD8 cells throughout the parenchyma and fibrous septa as well as around bile ducts in patients with liver disease. Biliary epithelial cells can secrete chemokines that localize lymphocytes to portal tracts.^5^, ^13^, ^33^, ^34^ We recently reported that VCAM‐1 on bile ducts supports effector T cells survival by binding to α4β1,^35^ but apoptosis pathways affecting different lymphocyte subsets in the inflamed liver are poorly understood. BECs express FASL, which can induce T cell death by activating Fas. Thus, expression or secretion of FASL by BECs could contribute to intrahepatic T cell apoptosis.^36^

Circulating T_reg_ are highly susceptible to CD95‐FASL‐dependent apoptosis but not to TCR‐mediated cell death in contrast to effector T cells.^11^ T_reg_ in the tumor undergo CD95‐dependent cell death as a consequence of FASL expression by tumor cells.^37^ We have reported previously that BECs express FASL in inflammatory liver diseases,^14^ but we have not explored the role of Fas in the differential fate of bile duct‐associated T_reg_ and CD8 cells. In the present study, we demonstrate that PEM T_reg_ are more susceptible to CD95‐FASL‐mediated apoptosis either in contact with BECs or in response to BEC supernatant compared with PEM CD8 cells. T_reg_ could be rescued by blocking FASL on BECs or in the inflamed biliary supernatant. If apoptosis of T_reg_ affects only those in contact with bile ducts, and not those elsewhere in the liver, this may not have a substantial effect on the overall frequency of T_reg_ in the diseased liver. In addition, the total frequency of T_reg_ in the inflamed liver will depend on other factors, including the balance between recruitment, retention, proliferation, and exit of cells from the liver.

IL‐2 is a crucial cytokine for T_reg_ survival that activates STAT5 phosphorylation in response to activation of the IL‐2 receptor. IL‐2 can selectively expand T_reg_ populations in vitro and in vivo.^38^, ^39^ We detected minimal IL‐2 in both the normal and inflamed liver microenvironment, which might account in part for reduced T_reg_ functional capacity in the inflammatory tissue. The intrahepatic source of IL‐2 is activated in CD4 and CD8 cells, which may be rapidly consumed by both effector and T_reg_ for their survival and proliferation.^40^, ^41^ Exogenous IL‐2 restored levels of STAT5 phosphorylation and protected T_reg_ from Fas‐mediated apoptosis but had no effect on CD8 T cells. Thus, the lack of IL‐2 in the inflamed liver microenvironment may result in unopposed Fas‐mediated T_reg_ apoptosis as well as contributing to defective intrahepatic LIT_reg_ function. Because the requirement for IL‐2 is much higher for CD8 cells, they may depend on different survival mechanisms. This is in agreement with a previous study on the effect of IL‐2 on peripheral blood lymphocyte subsets.^42^ This enhanced phosphorylation of STAT5 in T_reg_ compared with CD8 cells in response to IL‐2 occurred in the presence and absence of cytokine‐enriched inflamed hepatic supernatant, suggesting that the differential effect of IL‐2 on T_reg_ compared with effector cells is maintained in an inflammatory environment. This finding provides support for the therapeutic manipulation of LIT_reg_ by treating these patients with IL‐2 along with T_reg_ therapy in the future to maintain functional and surviving LIT_reg_.

In conclusion, we show for the first time that LIT_reg_ are phenotypically stable within the inflamed hepatic environment and exhibit minimal plasticity. They remain functionally suppressive but are susceptible to apoptosis via the Fas pathway mediated by FASL on bile ducts, and this can be inhibited by exogenous IL‐2. Therefore, IL‐2 therapy may successfully restore intrahepatic T_reg_ numbers and function in liver disease.

Author names in bold denote shared co‐first authorship.

## Supporting information

Additional Supporting Information may be found at onlinelibrary.wiley.com/doi/10.1002/hep.28517/suppinfo.

Supporting Information Figure S1

Supporting Information Figure S2

Supporting Information Figure S3

Supporting Information Figure S4

Supporting Information Figure S5

Supporting Information
