# Supplementary figures and images for: Human intrahepatic regulatory T cells are functional, require IL‐2 from effector cells for survival, and are susceptible to Fas ligand‐mediated apoptosis
Source: Hepatology. 2016 Apr 15;64(1):138–50. doi: 10.1002/hep.28517 (PMC4950043; doi:10.1002/hep.28517)

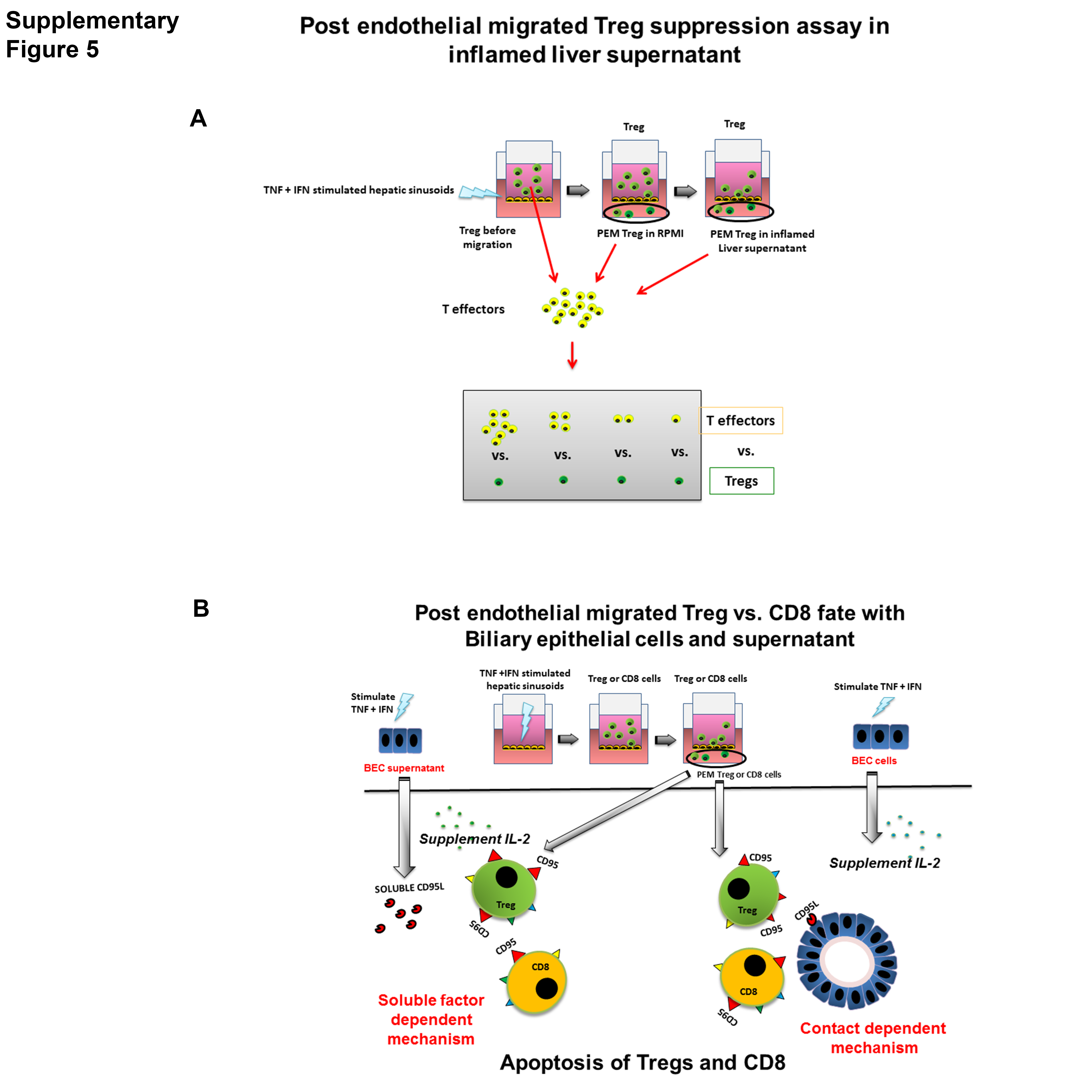

Supplement: Supplementary file 5 — Supporting Information Figure S5 [file HEP-64-138-s005.tif]
