# Supplementary material for: Human intrahepatic regulatory T cells are functional, require IL‐2 from effector cells for survival, and are susceptible to Fas ligand‐mediated apoptosis
Source: Hepatology. 2016 Apr 15;64(1):138–50. doi: 10.1002/hep.28517 (PMC4950043; doi:10.1002/hep.28517)
Supplement: Supplementary file 6 — Supporting Information [file HEP-64-138-s006.pdf]

Supplementary method

Isolation of peripheral blood regulatory T cells and CD8

Human peripheral blood mononuclear cells (PBMC) were isolated by density gradient centrifugation over Lympholyte (VH Bio, Gateshead, UK) at 800x*g* for 20 minutes. T_{reg} were isolated from PBMC using the $CD4^{+}CD25^{+}CD127^{dim/-}$ T_{reg} isolation Kit (Miltenyi Biotech) according to manufacturer's instructions and $CD8^{+}$ T cells were isolated from PBMC using the Dynabeads untouched human CD8 T cells kit (Life Technologies) according to manufacturer's instructions. Purity was greater than 98% as confirmed by flow cytometry.

Analysis of T_{reg} function, plasticity and response to IL-2 in the hepatic microenvironment

To examine T_{reg} suppressive ability, PEM T_{reg} were co-cultured with CellTrace™ violet (Life Technologies)-labelled $CD4^{+}CD25^{-}$ responder T cells (T_{resp}) at PEM T_{reg} to T_{resp} ratios ranging from 1:1 to 1:8. The Treg Suppression Inspector (Miltenyi Biotec) was added to the culture according to the manufacturer's instruction to stimulate T cell proliferation. T_{resp} proliferation was determined by flow cytometry on day 5.

Plasticity of PEM T_{reg} was also investigated by flow cytometry. Cells were stained for Th1, Th17 and T_{reg} transcription factors (Tbet (eBio4B10), RORc (AFKJS-9) and FOXP3 (PCH101) (all from eBioscience)) and intra-cellular cytokines (IFN-γ (45.B3, eBioscience), IL-17 (eBio64CAP17, eBioscience) and IL-10 (JES3-9D7, BD biosciences)) using the FoxP3/Transcription Factor Staining Buffer Set (eBioscience) according to the manufacturer's instructions.

To examine responsiveness of T_{reg} or CD8 cells to stimulation with IL-2, pre and post endothelial migrated cells were harvested into separate tubes and surface stained with FITC

anti-human CD127 (eBioRDR5, eBioscience) for 10 minutes at room temperature. Cells were then stimulated for 10 minutes at 37°C with or without 100 IU/ml Proleukinâ (aldesleukin). After washing with PBS cells were fixed for 12 minutes at 37°C with pre-warmed BD Phosflow buffer I (BD biosciences) and permeablised on ice for 30 minutes using pre-chilled (-20°C) BD Phosflow Buffer III (BD biosciences). After further washing with ice-cold PBS containing 2% foetal bovine serum (Sigma) (2%FCS-PBS) cells were stained for 1 hour at room temperature in 2% FCS-PBS with Alexa Fluor 647 anti-pSTAT5 (Y694) (47/Stat5 (pY694, BD Biosciences), PeCy7 anti-human CD3 (BD Biosciences), PerCP/Cy5.5 anti-human CD4 (RPA-T4, eBioscience), PE-CF594 anti-human CD8 (RPA-T8, BD Biosciences) and PE anti-human CD25 (3G10, Miltenyi biotec). All data were acquired using a Dako CyAn flow cytometer and analysed using Summit or Flowjo (Tree Star) software.

ELISA

Concentrations of IL-2 in cell culture supernatants recovered from human biliary epithelial cells, primary stellate cells, hepatic sinusoidal endothelial cells and hepatocytes that were unstimulated or stimulated with TNF-α (10ng/ml) or IFN-g (100ng/ml) and supernatants from isolated liver infiltrating $CD3^{+}CD4^{+}$ and $CD3^{+}CD8^{+}$ T cells that were unstimulated or stimulated with anti-CD3/anti-CD28 Dynabeads (Life Technologies) for 12 hours as well as cytokines IL6, IL8, IL12, IFN-γ, TNF-α, IL15, IL1β and IL-2 in explanted human liver supernatants were measured by ELISAs according to manufacturer's instructions. Liver supernatants were mostly prepared by culturing liver tissue at 1g/4ml RPMI overnight. For detection of IL-2 it was necessary to prepare higher concentration supernatants (1g or 2g tissue/1ml RPMI). Liver supernatants were filtered and remaining debris removed by centrifugation before loading onto the ELISA.

Immunohistochemistry

Immunohistochemistry staining was performed on 5µm formalin-fixed paraffin embedded sections after antigen retrieval. Peroxidase quenching with 0.3% H_2O_2 in methanol for 30 minutes was performed prior to microwaving at full power for 15 minutes in high pH antigen unmasking solution (Vector Labs). The sections were then washed in PBS and blocked with casein solution for 20 minutes at room temperature in a humidified chamber. Next slides were incubated at room temperature with anti-human CD8 (rabbit polyclonal antibody; 1:100, Abcam), anti-human CK19; b170; 1:100, Vector Labs), anti-human FAS Ligand (rabbit polyclonal antibody; 1:100, Abcam) or anti-FOXP3 (236A/E7; 1:20 or 1:80 (for dual staining) Abcam) for 1hour. The sections were then incubated with secondary antibody (Vector Impress Universal mouse/rabbit Ig kit, Vector Labs) and developed with DAB substrate (Vector labs). Sections for dual staining were then incubated at room temperature with casein solution for 20 minutes followed by anti-human caspase-3 (rabbit polyclonal antibody; 1:200, Abcam) for 1hour. After washing with TBS-0.1%tween, pH8.2 they were incubated with HRP-conjugated secondary antibody (Vector Impress rabbit Ig kit) for 30 minutes and developed with Vector Blue AP substrate (Vector Labs). Finally sections were counter stained with haematoxylin and mounted. Slides were examined using a Zeiss Axioskop40 microscope under 200x and 400x magnifications.

Confocal microscopy

Frozen liver tissue sections were blocked in casein and primary antibodies including anti-human CD8 (1:100, mouse IgG2b, clone (4B11) Vector Labs) and anti-human CK19 (1:100, mouse IgG1, clone b170 Vector Labs) applied for one hour followed by conjugated secondary antibodies AF555 anti-mouse IgG_{2b} (Life Technology), and FITC anti-mouse IgG_1 (Life Technology) for 30minutes. They were then incubated with DAPI (1µg/ml, Life Technology) to stain the cell nuclei. Between stains sections were washed for 10 minutes

with PBS. All incubation steps were performed in a humidified chamber protected from the light. Slides were visualized by confocal microscopy using LSM software (ZEISS).

TUNEL Assay on tissue sections

Apoptosis was detected by in situ end-labeling of fragmented DNA using TACS DAB In Situ Apoptosis Detection Kit (Trevigen, Inc.). Tissue sections were deparaffinized and pretreated with proteinase K solution for 5 minutes at room temperature. Endogenous peroxidase activity was blocked by 0.3 % H_2O_2 in methanol for 5 minutes. After pre-incubation in labeling buffer for 5 minutes, the specimens were incubated with brominated nucleotide (BrdU) and terminal deoxynucleotidyl transferase enzyme (TdT) for 1hour at 37°C. Following terminating the reaction by stop buffer, the specimens were incubated with anti-BrdU in Strep dilution for 30 minutes at 37°C. Then the sections were reacted with Strep-horseradish peroxidase for 10 minutes. DAB staining and haemotoxylin staining were applied before light microscopic examination.

Multicolour flow cytometry

Fresh LIL from different human diseased livers, normal liver and post endothelial migrated T_{reg} and CD8 were stained for surface markers of interest were phenotyped using APCH7 CD3, HorizonV500 CD4, PE CD44, BV421 CD25, PE anti CD154, PE anti CD134, APC CCR7 (all BD Biosciences), PerCPCy5.5 CD8 (Life Technologies), FITC CD127, PE CD26, PE CD39 PE CD69, PE PD1, PE CD178, PE CD40, PE CD95, PE CD27, PE IL-6R, PE IL-15R, PE CD45RA, PE CD45RO (all from eBioscience). For intracellular cytokine and transcription factor analysis, cells were activated with PMA and ionomycin for 4 hours, fixed and permeabilized using the BD Cytofix/Cytoperm™ Kit, then stained with PE anti-human IL10 (BD Biosciences), PE FOXP3, PE RORc, PE IL17, PE Tbet and PE IFNγ (all from

eBioscience). Data were acquired using a CyAn flow cytometer and analysed using Summit software.

Analysis of IL-2 production by liver infiltrating $CD4^+$ and $CD8^+$ T cells

LIL were isolated and separate populations of $CD3^+CD4^+$ and $CD3^+CD8^+$ T cells isolated by fluorescence activated cell sorting with a Moflo Astrios cell sorter (Beckman Coulter) after staining with BV421-anti-human CD3 (UCHT1, BD biosciences), Viogreen anti-human CD4 (VIT4, Miltenyi Biotec) and PerCPVio700 anti-human CD8 (BW135/80, Miltenyi Biotec). Cells were plated at $1x10^6$ cells/ml for 12hours in a round-bottomed 96-well plates and stimulated with or without anti-CD3/anti-CD28 coated Dynabeads (Invitrogen) at 4cells/bead. Supernatants were collected at 12hours and stored at -20°C for analysis of IL-2 cytokine production by ELISA using the human IL-2 ELISA Ready-Set-Go kit (eBioscience) according to manufacturer’s instructions. After removal of supernatants for measurement of IL-2 production the volume was replaced with fresh culture medium and cells stimulated with or without PMA and ionomycin in the presence of Brefeldin A for 4 hours. Frequencies of IL-2 producing cells were determined by flow cytometry. Cells were fixed with 3% formaldehyde after staining for dead cells with the fixable viability dye eFluor 506 (eBioscience) then stained for intracellular IL-2 using APC anti-human IL-2 (APC, eBiosciene) in 0.1% Saponin solution. Data were acquired using a CyAn flow cytometer and analysed using Flowjo (Tree Star) software.

Supplementary figure legends

Supplementary figure 1

(A) Peripheral blood T_{regs} were isolated from normal subjects and phenotyped for their expression of T_{reg} surface markers by flow cytometry. T_{reg} were gated as $CD4^{+}CD25^{+}CD127^{low}$. Data represent mean ± SEM, n=4._**(B)** T_{reg} were isolated from peripheral blood and transmigrated across TNF-α and IFN-γ-stimulated human sinusoidal endothelium into RPMI or inflamed liver supernatant. Post endothelial migrated cells and non-transmigrated cells were collected and expression of LFA-1 and VLA-4 on $CD4^{+}CD25^{+}CD127^{-}$ T_{reg} examined by flow cytometry. **(C)** Liver infiltrating CD8 cells from human explanted diseased livers and normal livers were phenotyped by flow cytometry for their expression of surface markers including: CD26, CD39, CD44, CD69 and PD1. Diseased livers are categorized as alcoholic liver disease (ALD, grey bar) and autoimmune diseased livers (black bar) and compared to normal livers (white bar). Data represent mean ± SEM, n=6 (One-way ANOVA followed by Bonferroni multiple comparison test, * $p < 0.05$.

Supplementary figure 2

Post-endothelial transmigrated T_{reg} (PEM T_{reg}) expression of transcription factors **(A)** FOXP3 **(B)** Tbet and **(C)** RORc at 24 hour intervals over the course of 72hours culture in inflamed liver supernatant. Freshly isolated T_{reg} were transmigrated through IFN-γ and TNF-α-stimulated hepatic sinusoidal endothelial cells into either RPMI-1640 (control) or liver supernatant (Supplementary Figure 5A). PEM T_{reg} were then analysed for expression of the T_{reg} transcription factor FOXP3, Th1 transcription factor Tbet and Th17 transcription factor RORc before migration (black bar), post

endothelial migration into RPMI (white bar) or post endothelial migration into inflamed supernatant (shaded bar) (n=4).

Supplementary figure 3

(A) Freshly isolated human liver infiltrating myeloid dendritic cells and plasmacytoid dendritic cells were defined by gating on (CD1c^{+} and CD11c^{+}) and (CD303^{+}CD123^{+}). We then assessed the expression of CD70 on both dendritic cell subsets (representative overlay histograms are shown). **(B)** Summary expression level of CD70 on human liver myeloid dendritic cells (grey bar) and plasmacytoid dendritic cells (black bar). Data are Mean ± SEM, n=4. **(C)** Freshly isolated peripheral blood CD4^{+}CD25^{+}CD127^{low} T_{reg} and CD8 cells were incubated with recombinant CD70 and their proliferation assessed over time up to 5 days by measuring the percentage of Ki67-positive cells in non-transmigrated cells (black bar) and cells transmigrated across stimulated human sinusoidal endothelium into RPMI (grey bar) or recombinant CD70 (white bar). **(D)** Immunohistochemistry staining showing the localisation of CD11c expressing dendritic cells (red) and FOXP3 expressing cells (Brown) in inflamed liver.

Supplementary figure 4

Peripheral blood mononuclear cells (PBMC) were transmigrated overnight across 24 hour TNF-α and IFN-γ-stimulated human sinusoidal endothelium into RPMI or inflamed liver supernatant. Post endothelial migrated cells and non-transmigrated cells were collected into separate fractions and stimulated for 10minutes with 0 or 100IU/ml IL-2. pSTAT5 (pY694) expression by CD4^{+}CD25^{+}CD127^{low} T_{reg} and CD8 cells in the fractions were examined by flow cytometry. The percentages of pSTAT5

expressing **(A)** T_{reg} and **(B)** CD8 cells were quantified. Data are Mean ± SEM, n=4. * P<0.05, ** P<0.01, *** P< 0.0001 by Paired *t*-test. Statistical tests compare untreated and IL-2 treated cells from the same fraction.

Supplementary Figure 5

Diagram illustrating the methods for in vitro analysis of **(A)** the effect of the inflamed liver microenvironment on the suppressive function of T_{reg} and **(B)** the fate of T_{reg} or CD8 cells in direct or indirect contact (soluble factor mediated) with human liver derived biliary epithelial cells. T_{reg} isolated from peripheral blood were transmigrated across TNF-α and IFN-γ-stimulated hepatic sinusoidal endothelial cells into RPMI (control media) or liver supernatant **(A)** or into culture with TNF-α and IFN-γ-stimulated biliary epithelial cells (BEC) or supernatants generated from stimulated BEC **(B)**. T_{reg} suppression assay **(A)** or analysis of apoptosis by annexin V staining **(B)** was performed on these transmigrated cells.
